# Comparative genome analysis and genome-guided physiological analysis of *Roseobacter litoralis*

**DOI:** 10.1186/1471-2164-12-324

**Published:** 2011-06-21

**Authors:** Daniela Kalhoefer, Sebastian Thole, Sonja Voget, Rüdiger Lehmann, Heiko Liesegang, Antje Wollher, Rolf Daniel, Meinhard Simon, Thorsten Brinkhoff

**Affiliations:** 1 Institute for Chemistry and Biology of the Marine Environment, University of Oldenburg, Carl-von-Ossietzky-Straße 9-11, 26129 Oldenburg, Germany; 2 Göttingen Genomics Laboratory, Institute of Microbiology and Genetics, Georg-August University of Göttingen, Grisebachstraße 8, 37077 Göttingen, Germany

## Abstract

**Background:**

*Roseobacter litoralis *OCh149, the type species of the genus, and *Roseobacter denitrificans *OCh114 were the first described organisms of the *Roseobacter *clade, an ecologically important group of marine bacteria. Both species were isolated from seaweed and are able to perform aerobic anoxygenic photosynthesis.

**Results:**

The genome of *R. litoralis *OCh149 contains one circular chromosome of 4,505,211 bp and three plasmids of 93,578 bp (pRLO149_94), 83,129 bp (pRLO149_83) and 63,532 bp (pRLO149_63). Of the 4537 genes predicted for *R. litoralis*, 1122 (24.7%) are not present in the genome of *R. denitrificans*. Many of the unique genes of *R. litoralis *are located in genomic islands and on plasmids. On pRLO149_83 several potential heavy metal resistance genes are encoded which are not present in the genome of *R. denitrificans*. The comparison of the heavy metal tolerance of the two organisms showed an increased zinc tolerance of *R. litoralis*. In contrast to *R. denitrificans*, the photosynthesis genes of *R. litoralis *are plasmid encoded. The activity of the photosynthetic apparatus was confirmed by respiration rate measurements, indicating a growth-phase dependent response to light. Comparative genomics with other members of the *Roseobacter *clade revealed several genomic regions that were only conserved in the two *Roseobacter *species. One of those regions encodes a variety of genes that might play a role in host association of the organisms. The catabolism of different carbon and nitrogen sources was predicted from the genome and combined with experimental data. In several cases, e.g. the degradation of some algal osmolytes and sugars, the genome-derived predictions of the metabolic pathways in *R. litoralis *differed from the phenotype.

**Conclusions:**

The genomic differences between the two *Roseobacter *species are mainly due to lateral gene transfer and genomic rearrangements. Plasmid pRLO149_83 contains predominantly recently acquired genetic material whereas pRLO149_94 was probably translocated from the chromosome. Plasmid pRLO149_63 and one plasmid of *R. denitrifcans *(pTB2) seem to have a common ancestor and are important for cell envelope biosynthesis. Several new mechanisms of substrate degradation were indicated from the combination of experimental and genomic data. The photosynthetic activity of *R. litoralis *is probably regulated by nutrient availability.

## Background

The genus *Roseobacter *comprises the two species *Roseobacter litoralis *OCh149 and *Roseobacter denitrificans *OCh114. Both species were isolated from marine seaweed and were the first described organisms of the *Roseobacter *clade [1]. *R. denitrificans *is able to grow anaerobically using nitrate or trimethyl-N-oxide (TMAO) as electron acceptors [1-3], whereas *R. litoralis *showed no denitrifying activity [1]. *R. denitrificans *and *R. litoralis *as well as some other members of the clade have the ability to use light energy and perform aerobic anoxygenic photosynthesis [1,4]. In *R. litoralis*, the photosynthesis genes are located on a plasmid, which is unusual for aerobic anoxygenic phototrophs (AAnPs) [5].

The genome sequences of more than 40 *Roseobacter *clade members are available, but only five of them are finished [6]. The genome sequence of *Roseobacter denitrificans *OCh114 was published in 2007 by Swingley and co-workers [7] and was the first genome of an aerobic anoxygenic phototrophic bacterium. The absence of ribulose bisphosphate carboxylase and phosphoribulokinase supports the assumption that AAnPs do not fix carbon dioxide via the Krebs-Cycle. Genes coding for other anaplerotic enzymes were found in the genome of *R. denitrificans *and the importance of mixotrophic growth was evident [7].

Plasmid-encoded functions are of great interest in genome analysis because plasmids often provide exchangeable niche specific fitness factors. Heavy metal resistances, e.g., are often encoded by plasmids [8,9] and are important for marine organisms as heavy metals accumulate in sediments [10,11], in macroalgae [12-14] but also in other aquatic organisms [15]. Consequently, many of the sequenced *Roseobacter *clade members harbour plasmids, but due to the fact that the majority of the sequences are not finished, not much is known about these plasmids. However, it is assumed that translocation processes between chromosomes and plasmids occur frequently [16].

The aims of our study were the genome characterization, comparative genomics and genome-guided physiological analysis of *R. litoralis*, the type strain of the genus *Roseobacter*. The genome of *R. litoralis *was compared to the genome of the closely related *R. denitrificans *as well as to 38 genomes of other members of the *Roseobacter *clade. Metabolic pathways were reconstructed and verified by physiological tests. Heavy metal tolerance tests with both *Roseobacter *species were performed to confirm differences of the species indicated by genomic data. Furthermore, insights into the regulation mechanism of the photosynthetic activity of *R. litoralis *are given.

## Results and discussion

General genomic features and comparison of the two *Roseobacter *species

**Figure 1:**
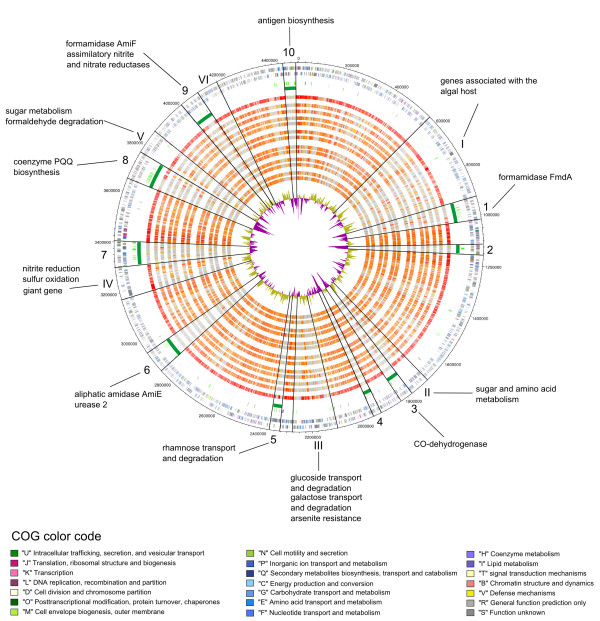
**Circular plot of the chromosome of *R. litoralis***. Rings from the outside to the inside: 1 and 2: open reading frames (ORFs) on the leading strand and on the complementary strand, respectively. Colours according to Clusters of Orthologous Groups (COG) -categories. 3: rRNA cluster (pink); 4: transposases (light green) and tRNAs (black); 5: genomic islands (dark green); 6-18: orthologous ORFs according to the Needleman-Wunsch-algorithm in the following organisms in the order of appearance: *Roseobacter denitrificans *OCh114, *Oceanibulbus indoliflex *HEL-45, *Sulfitobacter *NAS-14.1, *Dinoroseobacter shibae *DFL-12, *Jannaschia *sp. CCS1, *Phaeobacter gallaeciensis *DSM17395, *Ruegeria pomeroyi *DSS-3, *Roseovarius nubinhibens *ISM, *Roseobacter *AzwK-3b, *Octadecabacter arcticus *238, *Loktanella vestfoldensis *SKA53, *Maritimibacter alkaliphilus *HTCC2654, Rhodobacterales bacterium HTCC2150. Two organisms were chosen of each phylogenetic group outlined by Newton *et al*. [7]. The shade of red illustrates the value of the algorithm with red bars representing the ORFs with the best conformity to the respective ORFs of *R. litoralis *and the grey bars showing the ORFs that have no orthologs in the respective organism. 19: G+C-content of the chromosome of *R. litoralis *with violet areas below average and olive areas above average. Genomic islands (GEIs, labelled with Arabic numerals) and hypervariable regions (HVRs, labelled with Roman numerals) are separated by black lines. Special features within the GEIs and HVRs are outlined and are further discussed in the text.

**Figure 2:**
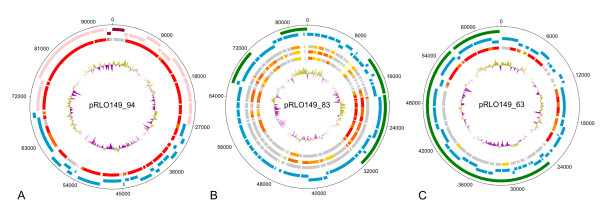
**Circular plots of the plasmids of *R. Litoralis***. **A-C: **The inner rings display the G+C-content with violet areas below average and olive areas above average. Orthologous ORFs in other organisms (red and grey bars) are according to the Needleman-Wunsch-algorithm. The shade of red illustrates the value of the algorithm with red bars representing the ORFs with the best conformity to the respective ORFs of *R. litoralis *and the grey bars showing the ORFs that have no orthologs in the respective organism. **A: **Plasmid pRLO149_94 of *R.litoralis*. Rings from the outside to the inside: 1 and 2: ORFs on the leading and complementary strands, respectively. Pink ORFs are associated with photosynthesis, blue ORFs have different functions, dark red ORFs show the replication genes of the plasmid. 3: orthologs in the genome of *R. denitrificans*. All orthologs can be found on the chromosome of *R. denitrificans *with ~60 kb being syntenic. Only nine ORFs do not have orthologs in the genome of *R. denitrificans *including the replication genes of the plasmid. **B: **Plasmid pRLO149_83 of *R. litoralis*. Rings from the outside to the inside: 1: predicted alien genes on the plasmid; 2 and 3: ORFs on the leading and complementary strands of the plasmid, respectively; 4-6: orthologous ORFs in *R. denitrificans*, *D. shibae *and *R. nubinhibens *in the order of appearance. Nearly all orthologs in the two latter organisms are also encoded on plasmids. **C: **Plasmid pRLO149_63 of *R. litoralis*. Rings from the outside to the inside: 1: predicted alien genes on the plasmid; 2 and 3: ORFs on the leading and complementary strands of the plasmid, respectively; 4: orthologous ORFs in *R. denitrificans*. The orthologs in the genome of *R. denitrificans *are all located on the 69 kb-plasmid of the organism. More than half of the plasmid encodes putative alien genes.

**Figure 3:**
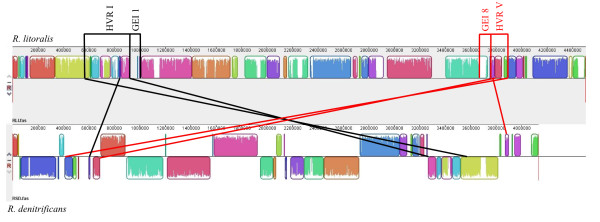
**Mauve alignment of the chromosomes of *R. litoralis *(upper line) and *R. denitrificans *(lower line)**. Colored boxes represent syntenic regions and white/grey areas the unique regions of the two organisms. The two *Roseobacter *species share a high amount of genetic material but the chromosomes have been subject to frequent rearrangements and events of lateral gene transfer. Marked are two HVRs and the adjacent GEIs of *R. litoralis *that have corresponding regions on similar positions of the chromosome of *R. denitrificans*. An X-like structure is formed when the HVRs are connected by lines. The areas display a mosaic-like structure of syntenic regions interspersed with unique regions in the respective organism. HVR I of *R. litoralis *contains a variety of genes that are connected to the association with the algal host of the bacterium, whereas HVR V contains genes for formaldehyde degradation and sugar metabolism. Both areas have little synteny with other *Roseobacter *clade members and are therefore important for the identification of genus-unique genes.

The manually curated and annotated final genome sequence of *R. litoralis *OCh149 comprises a chromosome with the size of 4,505,211 bp and three plasmids of 93,578 bp (pRLO149_94), 83,129 bp (pRLO149_83) and 63,532 bp (pRLO149_63), respectively (Table 1, Figures 1 and 2). The genome encodes 4537 predicted genes. The average G+C content of the genome is 57.23%. According to reciprocal BLAST analysis, the two *Roseobacter *species share a core genome consisting of 3415 genes (75.3% of the genes of *R. litoralis*). The chromosomes of the two organisms have been subject to many genomic rearrangements that are evident in the chromosomal alignment (Figure 3). Of the 1122 unique genes (24.7%) of *R. litoralis*, 226 are located on plasmids. In *R. denitrificans*, 714 (17.3%) genes are unique of which 148 are plasmid-encoded. Many of the unique genes on the chromosomes occur in genomic islands (GEIs), but a variety of species-specific genes are scattered over the chromosomes. According to Clusters of Orthologous Groups (COG) -categories, the majority of these genes are involved in amino acid and carbohydrate metabolism. The unique genes with assigned function of *R. litoralis *and *R. denitrificans *are listed in Additional File 1.

**Table 1:** General features of the genomes of *R. litoralis *and *R. denitrificans*

*R. litoralis*	chromosome RLO149c	pRLO149_94	pRLO149_83	pRLO149_63	
Size [bp]	4,505,211	93,578	83,129	63,532	
Protein coding sequences	4,311	86	93	47	
Pseudogenes	77			14	
G+C content [%]	57	58	59	55	
rRNA operons	1				
tRNAs	37				
***R. denitrificans***	**chromosome**	**pTB1**	**pTB2**	**pTB3**	**pTB4**

Size [bp]	4,133,097	106,469	69,269	16,575	5,824
Protein coding sequences	3,946	105	56	16	6
Pseudogenes	20	1			
G+C content [%]	58	55	59	55	55
rRNA operons	1				
tRNAs	38				

Data for *R. denitrificans *according to the NCBI database [96]

### Genomic islands

Ten GEIs were identified on the chromosome of *R. litoralis *(Figure 1, tagged with Arabic numerals) making up ~665 kb (14.8%). In *R. denitrificans*, in contrast, only ~300 kb (7.1%) were identified as genomic islands. The excess of 365 kb of alien genetic material in *R. litoralis *corresponds to the larger chromosome size (~372 kb, see also Table 1) of the organism. Thus, the additional genetic material of *R. litoralis *was most likely acquired via horizontal gene transfer.

Typically, GEIs contain a G+C content and a Codon Adaptation Index (CAI) different from the average [17]. Furthermore, transposases within the islands and tRNAs flanking the GEIs are indicators for translocation processes [17,18]. Many of the genes located in GEIs are of unknown function and several do not exhibit significant similarities to other genes in the databases (orphan genes). These orphan genes are thought to be phage-derived genetic material [19]. Although no complete prophages are present in the genome of *R. litoralis*, in some of the islands phage-like genes were identified, e.g. in island 8 three putative phage tail proteins are located (RLO149_c037250 - RLO149_c037270).

In other GEIs, however, genes were identified that were probably derived from other bacterial species. Frequently, amino acid and carbohydrate transport and metabolism genes are present in the GEIs, providing *R. litoralis *with additional abilities for substrate utilization. For example island 5 contains genes for rhamnose transport and degradation (RLO149_c023060 - RLO149_c023140) that have been described in Rhizobia [20]. Genes involved in nitrogen metabolism were identified in islands 1, 6 and 9 including different amidases (RLO149_c009550, RLO149_c040080, RLO149_c040170, RLO149_c028370), a second uncommon urease gene cluster (RLO149_c028310 - RLO149_c028360) and assimilatory nitrite and nitrate reductases (RLO149_c039830 - RLO149_c039850). Island 4 contains a carbon monoxide dehydrogenase encoding gene (CODH, RLO149_c017450 - RLO149_c017470), which is not present in *R. denitrificans*. The carbon monoxide dehydrogenase was believed to be common in the *Roseobacter *clade, as all members contain the corresponding genes [21-23]. However, recently it was shown that only a small proportion of *Roseobacter *clade members, among those *R. litoralis*, are able to oxidise carbon monoxide and that a wide variety lacks an essential subunit of the CODH-complex [24]. *R. denitrificans *is not able to oxidise carbon monoxide, but instead, has genes coding for a nitrate reductase that enables the organism to reduce nitrate under anaerobic growth conditions. Island 8 contains genes for coenzyme PQQ biosynthesis (RLO149_c036920 - RLO149_c036960) that are also located in a GEI of *R. denitrificans*. In island 10, genes for antigen biosynthesis were identified, e.g. UDP-N-acetylglucosamine 2-epimerase WecB (RLO149_c044390) and UDP-4-amino-4-deoxy-L-arabinose--oxoglutarate aminotransferase ArnB (RLO149_c044340). The latter is also similar to the perosamine synthetase from *Brucella melitensis*, with GDP-perosamine being part of the O-antigen of the organism [25]. Antigens are polysaccharides and lipopolysaccharides that define the structure of the bacterial cell surface. Genes important for cell envelope biosynthesis are often found in islands of environmental bacteria [26]. The cell surface structure is important for biofilm formation and host association of the organisms and structural alteration can provide niche adaption and phage defence [19,26-28].

### Unique genes on plasmids

Several species-specific genes, of which the majority is associated with heavy metal resistance (Table 2), are located on plasmid pRLO149_83. Therefore, the two *Roseobacter *species were compared with respect to zinc and copper tolerance. *R. litoralis *showed a higher tolerance of zinc, whereas *R. denitrificans *showed a higher copper tolerance. *R. litoralis *could grow without impairment up to 0.08 mM of zinc, but was inhibited in its growth in the presence of low copper concentrations (0.04 mM). In contrast, *R. denitrificans *could not grow with 0.02 mM zinc added to the medium, but was able to grow with 0.1 mM of copper. The higher zinc tolerance of *R. litoralis *could be due to the Zn-Cpx-type ATPase and/or the putative cobalt-zinc-cadmium resistance protein CzcD (Table 2), a member of the cation diffusion efflux (CDF) family [29]. Substrates of CDF proteins can be various cations [29], but mainly Zn^2+^-transporting CDFs such as ZitB from *Escherichia coli *[30] are also known.

**Table 2:** Heavy metal resistance genes encoded on plasmid pRLO149_83 of *R. litoralis*

**Accession No**.	Gene Name	Annotation	Metal Ions
**Copper oxidase systems**			

RLO149_p830810		copper resistance-like protein	Cu^2+^
RLO149_p830800		copper resistance-like protein	Cu^2+^
RLO149_p830790		putative copper resistance protein A	Cu^2+^
RLO149_p830650		copper resistance-like protein	Cu^2+^
RLO149_p830640		cupredoxin-like protein	Cu^2+^
RLO149_p830630		cupredoxin-like protein	Cu^2+^
**CPx-type ATPases**			

RLO149_p830740	*actP*	copper-transporting P-type ATPase (EC 3.6.3.4)	Cu^+^/Ag^+^
RLO149_p830520	*actP*	copper-transporting P-type ATPase (EC 3.6.3.4)	Cu^+^/Ag^+^
RLO149_p830380		cation transport ATPase (P-type) family	Zn^2+^
**HME-RND-proteins**			

RLO149_p830440		cation efflux system protein CusB-like protein	aerobically Ag^+^/anaerobically Cu^+^
RLO149_p830430	*cusA*	cation efflux system protein CusA	aerobically Ag^+^/anaerobically Cu^+^
RLO149_p830420	*cusF*	cation efflux system protein CusF	aerobically Ag^+^/anaerobically Cu^+^
**CDF**			

RLO149_p830240		putative cobalt-zinc-cadmium resistance protein CzcD	Zn^2+^
**Others**			

RLO149_p830340		putative ZIP Zinc transporter	Zn^2+^
RLO149_p830610		putative integral membrane protein DUF6	?

The proteins are categorized into different heavy metal efflux protein families. Based on sequence similarities the metal ions most likely transported by the efflux systems are indicated in column 4. HME-RND, Heavy Metal Efflux - Resistance-Nodulation-Cell division protein family; CDF, cation diffusion efflux proteins.

Most of the other putative heavy metal resistance genes on plasmid pRLO149_83 have weak similarities to known copper and silver efflux proteins (Table 2). But since no higher copper tolerance of *R. litoralis *compared to *R. denitrificans *was observed, the efflux systems might be involved in transport of other cations. Two other members of the *Roseobacter *clade, *Dinoroseobacter shibae *DFL-12 and *Roseovarius nubinhibens *ISM, have orthologous heavy metal resistance genes on their plasmids (Figure 2B).

Plasmids are important mobile genetic elements and therefore often contain recently acquired genetic material. Thus, the occurrence of species-specific and also alien genes on two of the plasmids (Figure 2B and 2C) was not surprising. Plasmid pRLO149_94, however, is an exception as no alien genes or genomic islands were identified on the plasmid. Nearly the entire genetic information of pRLO149_94 was found on the chromosome of *R. denitrificans*, with approximately 78 kb being syntenic. Also in *R. denitrificans *the area was not identified as GEI. Only nine ORFs on the plasmid are not present in the genome of *R. denitrificans*. The genes for the photosynthetic apparatus comprise ~45 kb and are part of the syntenic area with the plasmid replication genes located amidst the photosynthesis genes in *R. litoralis *(Figure 2A). The remaining 33 kb that are syntenic in both organisms are located upstream of the photosynthesis genes in *R. denitrificans *and encode other functions. The genes downstream of the syntenic region to plasmid pRLO149_94 in *R. denitrificans *are also present in *R. litoralis *and are located approximately 100 kb upstream of *dnaA*. At the position on the chromosome of *R. litoralis *that corresponds to the position of the first gene associated with the photosynthesis apparatus of *R. denitrificans *(*idi*, RD1_0147) several transposases are encoded (Figure 1, upstream of island 10), suggesting this region to be a genomic hot spot. These findings suggest a translocation of the genetic material from the chromosome to the plasmid. This is further supported by the fact that photosynthesis genes are usually located on the chromosomes of AAnPs [5] and are thought to be rather vertically than horizontally acquired genetic material in *Roseobacter *clade bacteria [23].

### Comparison with other members of the *Roseobacter *clade

To identify the genes specific for the genus *Roseobacter*, the genomes of both species were compared to 38 genomes of other *Roseobacter *clade bacteria. The results of the comparison are shown for two representatives of each phylogenetic subgroup of the *Roseobacter *clade outlined by Newton et al. [22] in Figure 1. Six hypervariable regions (HVR I-VI), areas of low conservation in the *Roseobacter*-clade, were found adjacent to the genomic islands predicted on the chromosome of *R. litoralis *(Figure 1). The HVRs of *R. litoralis *are characterized by a mosaic-like structure, with regions conserved in all *Roseobacter *clade bacteria alternating with genus-unique but also species-unique genes. Frequently, tRNAs were found flanking the HVRs, but not many transposases were present inside the areas indicating that the regions are permanently anchored in the chromosome [31]. The G+C-content and also the codon-adaptation index vary inside the HVRs.

Of special interest is HVR I as many genes identified in this area seem to be connected to the relation of *R. litoralis *to the algal host. For example, several genes for the degradation and transport of algal osmolytes like taurine (RLO149_c007790 - RLO149_c007880) and sarcosine (RLO149_c006600 - RLO149_c006630) were identified in HVR I. Furthermore, the genes for vitamin B12 biosynthesis (RLO149_c006160 - RLO149_c006260) are present. Vitamin B12 was shown to be important for the symbiosis of *D. shibae *with its dinoflagellate host [32]. A degradation pathway of erythritol is also located in HVR I (RLO149_c008260 - RLO149_c008410). The pathway is only present in *R. litoralis *and *R. denitrificans *and is known from Rhizobia [33,34]. In *Rhizobium leguminosarum *erythritol catabolism is important for competitiveness of the organism in the nodulation of pea plants [34]. Thus, erythritol catabolism might also be associated with the algal host relation of the *Roseobacter *species. The mosaic-like structure of HVR I resembles the symbiosis islands of Rhizobia [18,35]. Two different areas can be identified on the chromosome of *R. denitrificans *that correspond to HVR I of *R. litoralis*. In the alignment of the chromosomes of the two species, the HVRs of the two organisms form an X-like structure (Figure 3) which is probably due to the tendency of genes in closely related organisms to be located in the same distance from the origin [36].

Uncharacterized sugar and amino acid transporters are frequently found in the HVRs, e.g. in HVRs II and V. Genes for glucoside (RLO149_c021710 - RLO149_c021770) and galactose/arabinose (RLO149_c021930 - RLO149_c021980) transport and degradation as well as an arsenite resistance system (RLO149_c022030 - RLO149_c022040) are located in HVR III. The genes for sulfur oxidation (RLO149_c031760 - RLO149_c031920) and the denitrification genes (RLO149_c031300 - RLO149_c031570) were identified in HVR IV. Due to the lack of the nitrate reductase in *R. litoralis *the organism is not able to reduce nitrate; however, genes encoding all other enzymes required for denitrification are present. Thus, it is possible that the organism is able to reduce nitrite to molecular nitrogen under anoxic conditions. HVR V contains genes for formaldehyde degradation that are more common in the mixotrophic than in the heterotrophic group of *Roseobacter *clade members [22].

### *rfb*-genes and host association

A *rfb-*gene cluster essential for the development of O-antigens, i.e. lipopolysaccharides of the outer membranes of Gram-negative bacteria [37], was identified on plasmid pRLO149_63 of *R. litoralis*. Many of the *Roseobacter *clade bacteria have *rfb*-genes in their genomes and in about half of those the genes are located on plasmids. In *R. denitrificans *the *rfb-*gene cluster is encoded on a plasmid of 69 kb (pTB2), a size similar to pRLO149_63. Approximately 50% of the genes on pTB2 and pRLO149_63 are orthologs (Figure 2C). The remaining parts contain unique genes for each organism, and many of these are associated with cell envelope biosynthesis. These findings suggest that the plasmids are important for the cell surface structure of the two *Roseobacter *species and may have originated from a common ancestor.

In *E. coli *the O-antigens are known to interact with the host defences and are therefore important virulence factors of pathogenic bacteria [37]. Many other *Roseobacter *clade bacteria with plasmid-located *rfb*-genes were also isolated from surfaces of algal or animal hosts. Therefore, we investigated whether a correlation between the replicon location of the *rfb-*genes and host-association exists. The genome sequences of marine *Rhodobacterales *species available in the IMG database [38,39] were searched for *rfb*-genes and the replicon location was determined if possible (see Additional File 2). For the unfinished genomes, co-occurrence of plasmid replication genes with the *rfb*-genes on the same DNA-contig was regarded as an indicator for plasmid localisation. Chromosome location was confirmed by rRNA clusters or chromosome partitioning genes on the DNA-contigs that harbour the *rfb*-genes. In 24 of the 39 genomes *rfb*-genes were present and their location could be identified. Chromosomal *rfb*-genes were found in 12 organisms and 11 of those were isolated from the water column. Nine of the 12 plasmid-located *rfb*-gene clusters were found in organisms that were isolated either from host surfaces, aquacultures, algae blooms or the like. Thus, the replicon analysis of the *rfb*-genes of 20 of the 24 organisms supports the hypothesis that plasmid-location of the *rfb*-genes is coherent with host-association of *Roseobacter *clade bacteria.

### Photosynthesis

**Figure 4:**
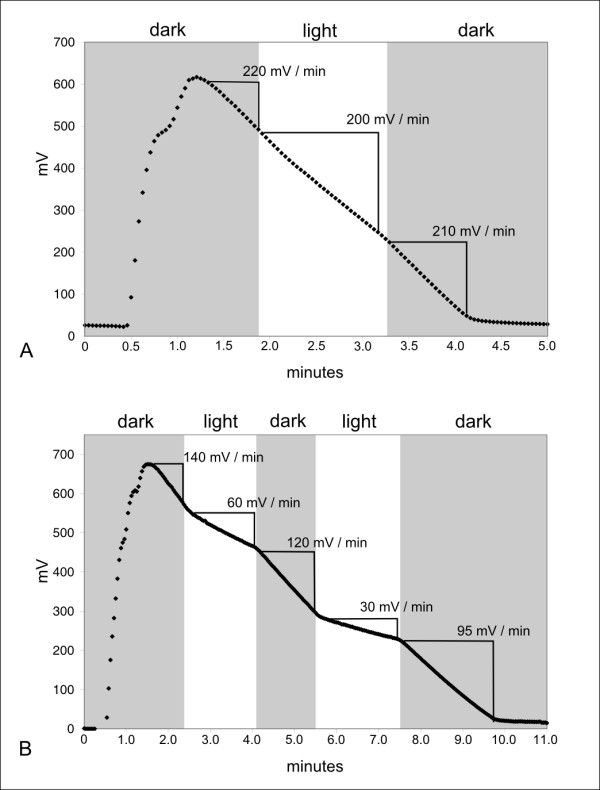
**Respiration rates of *R. litoralis *cells**. **A: exponential growth phase; B: stationary growth phase.** The values in mV min^-1 ^indicate the respiration rates in the respective time intervals. Cells were kept anoxic under nitrogen gas until oxygen was supplied. At the beginning of each respiration measurement the cell suspension was saturated with oxygen and the oxygen consumption of the cells was measured in mV min^-1^. The response of *R. litoralis *to light differs remarkably between the two growth phases. During the exponential growth phase the initial respiration rate in the dark was higher (220 mV min^-1^) than in the stationary growth phase (140 mV min^-1^). When exposed to light, the cells that were in the exponential growth phase showed only a slight decrease to 200 mV min^-1 ^(10%) of the respiration rate whereas in the stationary phase culture the respiration rate was reduced to 43% (60 mV min^-1^) of the original rate in the first light period and to 25% (30 mV min^-1^) in the second. Cells resumed 95% (exponential growth phase) and 86% (stationary growth phase) of their original respiration rate when darkened again.

To confirm the functionality of the plasmid-encoded photosynthesis apparatus in *R. litoralis*, the photosynthetic activity of the strain was measured via oxygen consumption (Figure 4). Whereas almost no reaction to light was observed during growth, cells in the stationary growth phase were highly responsive to light and showed a reduced respiration rate when exposed to light (Figure 4). Even though pigmentation occurred already during the exponential growth phase, the organism did not use the photosynthetic apparatus until the culture reached the stationary growth phase. The use, but not the expression, of the photosynthesis apparatus might therefore be influenced by nutrient availability in *R. litoralis*, as stationary phase cultures are nutrient depleted. We obtained similar results for *R. denitrificans*, with cells in the late stationary phase showing a stronger response to light than cells from the exponential growth phase (data not shown). For the alpha-*Proteobacteria Labrenzia alexandrii *DFl-11 and *Hoeflea phototrophica *DFL-43 periodic nutrient starvation has been reported to trigger bacteriochlorophyll-*a *production whereas only slight effects were observed for *D. shibae *[40]. Obviously, the regulation mechanisms differ between the aerobic phototrophic bacteria and so does the architecture of their photosynthesis genes. In Additional File 3, the organization of the photosynthesis gene clusters of the organisms mentioned above is compared, showing that organisms with similar physiological traits also have similar gene organizations. The suggestion that the organization of genes within purple bacterial photosynthesis gene clusters reflects regulatory mechanisms, evolutionary history, and relationships between species was also made by other authors [7,41,42]. In the oligotrophic environment of the ocean, the use of the photosynthesis apparatus during nutrient depletion may be an important advantage for *Roseobacter *species in the competition with non-photosynthetic organisms.

### Substrate tests and pathway reconstruction

**Figure 5:**
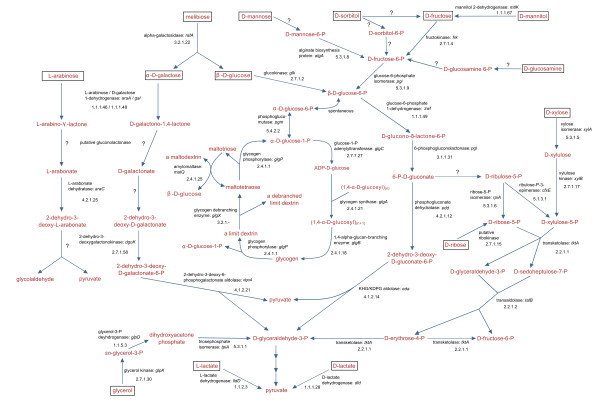
**Predicted glycogen metabolism and catabolic pathways of sugars and sugar derivatives degraded by *R. litoralis***. The substrates that were used by *R. litoralis *as carbon sources in the experiments are framed. Metabolites are shown in red, enzyme and gene names as well as the EC numbers in black. If available, the corresponding gene names of the enzymes are given. Question marks indicate that no genes for the respective reaction were predicted from the genome of *R. litoralis*. If question marks are combined with enzyme names, the genes were not unambigously annotated and the given enzyme is proposed to be involved in the reaction.

**Figure 6:**
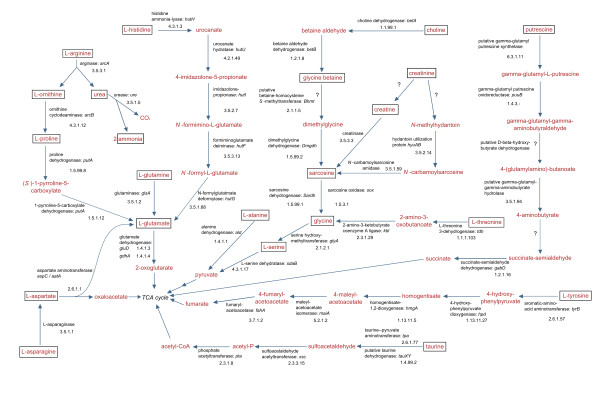
**Predicted catabolic pathways of amino acids, amino acid derivatives and algal osmolytes degraded by *R. litoralis***. The substrates that were used by *R. litoralis *as carbon sources in the experiments are framed. Metabolites are shown in red, enzyme and gene names as well as the EC numbers in black. If available, the corresponding gene names of the enzymes are given. Question marks indicate that no genes for the respective reaction were predicted from the genome of *R. litoralis*. If question marks are combined with enzyme names, the genes were not unambigously annotated and the given enzyme is proposed to be involved in the reaction.

Growth on different substrates was tested for *R. litoralis *to substantiate the reconstruction of metabolic pathways based on the genomic analyses. The results of the growth experiments are shown in Additional File 4. For each substrate the growth characteristics of *R. litoralis *and the genomic data were combined and the putative degradation pathways for the substrates allowing growth are shown in Figures 5 and 6. For the use of amino acids and amino acid derivatives, the predictions from the genome are mainly consistent with the experimentally achieved data (Additional File 4). However, for the degradation of sugars, sugar derivatives and algal osmolytes, genomic and experimental data differ in several cases (Additional File 4). Selected examples are discussed below.

### D-mannose and D-glucosamine

For D-mannose and D-glucosamine the process of phosphorylation could not be revealed from the genomic data (Figure 5). In other bacteria, a phosphotransferase system (PTS) mediates phosphorylation of monosaccharides already during transport (for reviews see [43,44]). As for the majority of the *Roseobacter *clade members, no complete PTS is encoded in the genome of *R. litoralis*. Present are genes for an EIIA component, Hpr and HprK, but no permease was identified. The function of the incomplete PTS is to date unknown. It rather exhibits a regulatory function as proposed for the spirochaetes *Treponema pallidum *and *Treponema denticola *[45] and as shown for *Sinorhizobium meliloti *[46], which also lack the permease component of the PTS. Therefore, a PTS-independent system for transport and phosphorylation of D-mannose and D-glucosamine must be present in *R. litoralis*.

### D-galactose, L-arabinose and D-fucose

*R. litoralis *grew with D-galactose, L-arabinose and D-fucose, but no complete pathways could be assigned to the degradation of these monosaccharides. A known mechanism for D-galactose degradation in bacteria is the DeLey-Doudoroff pathway (DD-pathway, [47]). Parts of the DD-pathway are encoded in the genome of *R. litoralis*, namely the genes coding for 2-dehydro-3-deoxygalactokinase (DgoK, RLO149_c015740) and 2-dehydro-3-deoxy-6-phosphogalactonate aldolase (DgoA, RLO149_c015730). The other genes required for the degradation of D-galactose via the DD-pathway are not present in this gene cluster (cluster 1, see Table 3). However, a gene with high similarity to galactose 1-dehydrogenase (Gal, RLO149_c021980) that catalyzes the first step of the pathway [47] was found elsewhere in the genome (cluster 2, see Table 3). This protein has also a high similarity to L-arabinose 1-dehydrogenase of *Azospirillum brasilense*. In this organism a pathway for L-arabinose degradation was described that is analogue to the DD-pathway for galactose degradation [48-50]. Beside L-arabinose 1-dehydrogenase, also arabonate dehydratase of *A. brasilense *has an ortholog in *R. litoralis *(RLO149_c021970) which is also located in cluster 2 (Table 3). The other proteins of the pathway known in *A. brasilense *cannot be assigned to proteins of *R. litoralis*. Thus, both pathways are incomplete in *R. litoralis*; however, if the two clusters are combined, a degradation mechanism based on the DD-pathway may be functional for both sugars (Figure 5). Correspondingly, the regulators as well as the sugar-binding periplasmic protein of the transport system in cluster 2 are known to interact with D-galactose, L-arabinose and D-fucose [51-54], and also other enzymes are known to act on all three sugars [55,56]. As no mechanism for D-fucose degradation was identified in the genome of *R. litoralis *and only weak growth was observed with this sugar, it is possible that the enzymes also act on D-fucose but with a lower affinity.

**Table 3:** Putative galactose degradation gene clusters in *R. litoralis*

**Accession No**.	Annotation
**Cluster 1**	

RLO149_c015710	BgaB: beta-galactosidase 1 (EC 3.2.1.23)
RLO149_c015720	putative gluconolactonase
RLO149_c015730	DgoA: 2-dehydro-3-deoxy-6-phosphogalactonate aldolase (EC 4.1.2.21)
RLO149_c015740	DgoK: 2-dehydro-3-deoxygalactonokinase (EC 2.7.1.58)
RLO149_c015750	short chain dehydrogenase
RLO149_c015760	RafA: alpha-galactosidase (EC 3.2.1.22)
RLO149_c015770	putative galactoside ABC transporter inner membrane component
RLO149_c015780	putative galactoside ABC transporter inner membrane component
RLO149_c015790	putative extracellular galactoside-binding protein
RLO149_c015800	HTH-type transcriptional regulator, IclR family
RLO149_c015810	putative galactoside ABC transporter ATP-binding protein
**Cluster 2**	

RLO149_c021930	putative HTH-type transcriptional regulator gbpR (Galactose-binding protein regulator)
RLO149_c021940	SbpA: multiple sugar-binding periplasmic protein
RLO149_c021950	putative multiple sugar transport ATP-binding protein
RLO149_c021960	putative multiple sugar transport permease protein
RLO149_c021970	AraC: L-arabonate dehydratase (EC 4.2.1.25)
RLO149_c021980	Gal: D-galactose 1-dehydrogenase (EC 1.1.1.48)

### Glycogen

*R. litoralis *was not able to grow with glycogen as a carbon source, probably due to the fact that no genes exist in the predicted secretome coding for extracellular glycogen cleavage enzymes, and also no putative transporters for glycogen were identified. Intracellular glycogen, however, was detected in cells of both *Roseobacter *species when grown in mineral medium. In contrast, no glycogen formation was observed when the cells were grown in complex medium. The glycogen biosynthesis and degradation genes in the two organisms are therefore probably involved in intracellular glycogen production under limiting conditions. The proposed pathway for glycogen metabolism in *R. litoralis *is shown in Figure 5.

Glycogen formation is often induced by nitrogen starvation [57-59]; however, both *Roseobacter *species were not nitrogen-starved. Thus, another limiting factor must be the inducer of glycogen production in the mineral medium.

In contrast to the other *Roseobacter *clade members, in both *Roseobacter *species the genes for glycogen biosynthesis and degradation are co-located with essential genes of the ED-pathway. It is known from other studies that *Roseobacter *clade members use the ED-pathway for sugar breakdown [60,61]. The co-location of the genes coding for the ED-pathway with those of the glycogen biosynthesis and degradation indicates a close relation of the central carbon metabolism and glycogen storage in the *Roseobacter *species.

### Algal osmolytes

The algal osmolytes tested in this study all served as carbon and nitrogen sources for both *Roseobacter *species, except for dimethylglycine. Additionally, *R. litoralis *used taurine as sulfur source. It was possible to reconstruct the pathways for algal osmolyte degradation from the genome of *R. litoralis *with the three exceptions creatinine, glycine betaine and putrescine (Figure 6, Additional File 4).

### Creatinine

Whereas the degradation mechanism for creatinine is clear in *R. denitrificans*, in *R. litoralis *the enzyme encoding the initial step of the pathway is not encoded in the genome (Figure 6). Nevertheless, no differences were observed between the two organisms when grown on creatinine. Furthermore, the enzymes mediating the second step of both possible pathways for creatinine degradation are present in the genome of *R. litoralis *(Figure 6). Thus, the organism is able to degrade creatinine, but the mechanism cannot be completely reconstructed from the genome.

### Glycine betaine

The gene coding for the enzyme converting glycine betaine to dimethylglycine (betaine-homocysteine S-methyltransferase, BHMT) was not identified in the genomes of the two *Roseobacter *species. However, a potential candidate gene is RLO149_c039100 which is co-located with the genes for dimethylglycine dehydrogenase (DMGDH4, RLO149_c039110) and a sarcosine dehydrogenase (SARDH, RLO149_c039090) that are both directly involved in osmolyte degradation (Figure 6) and are annotated according to eukaryotic enzymes. The domain homocysteine S-methyltransferase (InterPro database [62], entry IPR003726) that was identified in the protein sequence of RLO149_c039100 is known to be part of mammalian BHMTs [63], but the predicted protein of *R. litoralis *shares only 26% identical amino acids with the mammalian enzymes (E-value 6e-09). A similar ORF (RD1_0018, 96% identity to RLO149_c039100) is encoded in the genome of *R. denitrificans *that is located in the same genomic neighbourhood as RLO149_c039100.

Only eight bacterial enzymes annotated as BHMT are present in the UniProt database [64]. An enzyme of *S. meliloti *has been described to mediate this step in glycine betaine degradation [65], but the protein sequence of RLO149_c039100 shows only weak similarity to this protein. The genomic location, the functional domain and the fact that both *Roseobacter *strains are able to grow with glycine betaine as carbon and nitrogen sources point to RLO149_c039100 and RD1_0018 being involved in the degradation of glycine betaine in *Roseobacters*, representing a new class of bacterial BHMTs.

### Dimethylglycine

Even though several genes coding for dimethylglycine dehydrogenases (DMGDH) were identified in the genomes of both *Roseobacter *species, the strains were neither able to utilize dimethylglycine (DMG) as carbon nor as nitrogen source. It is possible that the DMGDHs are only mediating the degradation of intracellular DMG derived from the cleavage of glycine betaine.

### Putrescine

The degradation of the osmolyte putrescine is likely to occur via 4-aminobutyrate, as some of the genes of the pathway are present (Figure 6). However, the enzymes mediating step three and four of the pathway were not predicted in the genome of *R. litoralis *and also the aminotransferase responsible for the last step of the pathway, i.e. the degradation of 4-aminobutyrate to succinate-semialdehyde, was also not identified. Steps three and four might be mediated by RLO149_c025400 (putative gamma-glutamyl-gamma-aminobutyrate hydrolase) and RLO149_c025390 (putative D-beta-hydroxybutyrate dehydrogenase). The last step of the pathway might be carried out by one of the uncharacterized aldehyde aminotransferases encoded in the genome of *R. litoralis*.

### Taurine

Two different pathways are postulated for the transport and degradation of taurine by microorganisms [66]. Apparently both pathways with different transport systems are present in *R. litoralis*. A taurine TRAP transport system in combination with a taurine dehydrogenase (TDH) also occurs in *R. denitrificans, D. shibae, Rhodobacter sphaeroides *and *Paracoccus denitrificans*, whereas a taurine ABC transporter and a taurine:pyruvate aminotransferase (Tpa) are present in most of the other genome sequenced *Roseobacter *clade members. Only *D. shibae*, *R. denitrificans *and *R. litoralis *have both uptake and degradation systems. Common features of these three organisms are their photosynthetic activity and the association with algal hosts [1,4,32,67,68]. The common feature of all taurine TRAP transporter-containing organisms is their ability to grow anaerobically [2,3,69-71]. The exception is *R. litoralis*, for which no anaerobic growth was reported yet. Nevertheless, the ability to grow anaerobically is indicated by the presence of genes for nitrite reduction (see above) and for dimethyl sulfoxide (DMSO)/TMAO reductases (RLO149_c007970, RLO149_c001820-RLO149_c001840). Anoxic conditions can occur during the collapse of algal blooms [72] which might also be the situation when high amounts of taurine and other algal osmolytes become available.

## Conclusions

Our results show that the differences between the two *Roseobacter *species and the larger chromosome of *R. litoralis *are mainly due to events of horizontal gene transfer. Furthermore, the genomes have been subject to numerous genomic rearrangements. Plasmid pRLO149_94 of *R. litoralis*, on which the photosynthesis genes are encoded, was most likely translocated from the chromosome as it can almost completely be found on the chromosome of *R. denitrificans*. The photosynthetic activity of *R. litoralis *was shown to be growth-phase dependent. Whereas almost no reaction to light was observed during exponential growth, the organism was highly responsive to light during stationary growth phase. These results suggest a regulation of the photosynthetic activity according to nutrient availability that might also be reflected in the genetic organization of the photosynthesis genes. A plasmid with partial synteny to pRLO149_63 is present in *R. denitrificans *indicating a common ancestor of the two plasmids. Both plasmids and 11 other plasmids of *Roseobacter *clade bacteria harbour *rfb*-genes. The majority of these organisms were isolated from animal or algal hosts suggesting a coherence of plasmid location and host association. New mechanisms for sugar and algal osmolyte degradation were indicated. The ability to store intracellular glycogen as well as the utilization of algal osmolytes was reported for the first time for *Roseobacter *clade bacteria. Several pathways could not be fully elucidated, indicating *R. litoralis *to employ alternative enzymes compared to the known reference organisms.

## Methods

### Genome sequencing and finishing

The strains *R. litoralis *OCh149 and *R. denitrificans *OCh114 were obtained from the German Collection of Microorganisms and Cell Cultures (DSMZ, Braunschweig Germany). The genome sequencing of *R. litoralis *was carried out at the J. Craig Venter Institute (Rockville, MD, USA) within the Microbial Genome Sequencing Project by a Sanger sequencing-based approach. For details see the Microbial Genome Sequencing Project [73]. The Sanger-based sequencing resulted in 7.97 coverage of the genome sequence. Gap closure and all manual editing steps were carried out at the Göttingen Genomics Laboratory (University of Göttingen, Germany) using the Gap4 (v 4.11) software of the Staden package [74]. Remaining gaps in the sequences were closed by primer walking on PCR products. Sequences were obtained using the Big Dye 3.0 chemistry (Applied Biosystems), customized sequencing primers and ABI3730XL capillary sequencers (Applied Biosystems).

### Gene prediction and annotation

The prediction of coding sequences (CDS) or open reading frames (ORFs) was done with YACOP [75]. The results were verified and improved manually by using criteria such as the presence of a ribosome-binding site, GC frame plot analysis, and similarity to known protein-encoding sequences using the Sanger Artemis tool [76]. Functional annotation of all ORFs was carried out with the ERGO software package [77] (Integrated Genomics, Chicago, IL, USA). The protein sequences of the predicted ORFs were compared to the Swiss-Prot and TrEMBL databases [78]. Functional domains, repeats and important sites were analysed with the integrated database InterPro using the Web-based tool InterProScan [79].

### Comparative genomics

The genomes of 38 *Roseobacter *clade members used for the comparison are the same as those used for the analysis of the *rfb*-genes and are listed in Additional File 2. Additionally, *Roseobacter *R2A57 and *Loktanella *sp. SE62 were included in the comparison. For comparative analysis, the BiBag software tool for reciprocal BLAST analyses as well as a global sequence alignment using the Needleman-Wunsch algorithm (pers. comm. Antje Wollherr and Heiko Liesegang, G2L Göttingen) was used. ORFs were considered as orthologs at a Needleman-Wunsch similarity-score of at least thirty percent and an E-value < 10e-21. Circular plots of DNA sequences were generated with the program DNAPlotter [80]. The comparative visualisation of the genetic organization of the photosynthesis genes of different organisms was realized with the GenVision software (DNASTAR, Inc., Madison, WI, USA). Whole genome alignments were performed and visualized with the Mauve Software Tool [81].

### Sequence analysis

The programs IslandViewer [82] and COLOMBO [83] were used for the detection of alien genes and genomic islands in the genomes of *R. litoralis *and *R. denitrificans*. To complete the computational prediction, the predicted regions were manually checked for elements commonly associated with GEIs like transposases, integrases, insertion sequence (IS)-elements, tRNAs and GC-content deviations [84]. For the prediction of the secretome of *R. litoralis*, PrediSi [85] was used.

The Codon Adaptation Index (CAI) measures the synonymous codon usage bias for a given DNA sequence by comparing the similarity between the synonymous codon usage of a gene and the synonymous codon frequency of a reference set. For the calculation of the CAI values the CAIcal server was used [86]. For the functional categorization of gene products, a BLAST search with all coding sequences was performed against the COG database [87].

The metabolic pathways were reconstructed with the Pathway Tools Software [88,89] from the BioCyc Database collection [90]. The pathways were manually curated if necessary.

### Measurement of photosynthetic activity

*R. litoralis *was cultured in 500 mL Erlenmeyer flasks containing 200 mL of modified PPES-II medium [1] (see Additional File 5). Cells were grown at 25°C on a rotary shaker at 120 rpm with a natural day-night-rhythm. After 40 hours of incubation (within the exponential growth phase) and 95 hours (stationary growth phase), respectively, the cultures were harvested by centrifugation (7000 rpm, 10°C, 15 minutes) and washed once with 100 mL of a salt solution containing 20 g/L NaCl, 13 g/L MgCl_2 _× 6 H_2_O and 11.18 g/L KCl. After an additional centrifugation step, the cell pellets were resuspended in 5 mL of the salt solution and the OD_600 _was adjusted to 10. Respiration of the cells was measured via oxygen consumption [4].

### Heavy metal resistance tests

The heavy metal resistance tests were carried out on agar plates based on a modified medium described by Shioi [91] (see Additional File 5). Heavy metal stock solutions were prepared as aqueous solutions of 50 mM CuCl_2 _× 2 H_2_O and O_4_SZn × 7 H_2_O, respectively, and sterile filtrated. The stock solutions were added to the autoclaved medium directly before pouring the plates. Each agar plate contained 20 mL of medium and 0.02, 0.04, 0.06, 0.08 or 0.1 mM of one of the heavy metals. Tests were prepared in duplicates. Agar plates containing no heavy metals served as controls.

The pre-cultures were grown as follows: single colonies of the strains were transferred from agar plates to 20 mL of modified 70% Marine Broth medium (MB, Difco 2216, see Additional File 5) in 100 mL Erlenmeyer flasks. Cells were grown at 22°C on a rotary shaker (80 rpm) with a natural day-night-rhythm to an optical density (600 nm) between 0.3 and 0.4. The cell suspensions were diluted 10^-4 ^fold and 800 μL of the dilution were plated on each agar plate containing zinc or copper.

### Substrate tests

Substrate tests were performed with *R. litoralis *to compare the experimental data with the genomic information. As genome comparisons of the two *Roseobacter *species revealed some putative differences, growth on algal osmolytes was tested for both organisms. All substrates tested as carbon, nitrogen or sulfur sources are listed in Additional File 4. Cells of *R. litoralis *and *R. denitrificans *were grown in sterile 22.5 mL metal-capped test tubes containing 5 mL of modified Marine Basal Mineral (MBM) medium [92] (see Additional File 5). Tests for taurine as sulfur source were carried out in modified MBM medium prepared without sulfate but with 100 μM taurine. For the substrates that were tested as nitrogen sources, Tris-HCl in the modified MBM medium was replaced by 0.19 g/L NaHCO_3 _and no NH_4_Cl was added. The pH of the medium was adjusted to 7.5 after autoclaving with sterile 100 mM HCl. Aqueous stock solutions of the substrates were prepared, sterile filtrated and stored at -20°C or at 4°C. Final concentrations for sugars, sugar derivatives, ethanol, glycogen and urea were 1 mg/mL, 2 mM for amino acids and amino acid derivatives, 10 mM for the algal osmolytes and 1 mL/L for glycerol. When the substrates served as nitrogen sources, the final nitrogen concentration was adjusted to 2.5 mM. All tests were carried out in parallels, one additional parallel was not inoculated and served as a control. For all substrates that were tested as carbon sources for *R. litoralis*, two additional parallels were supplemented with 1% of complex medium (modified 70% MB, see Additional File 5) to investigate whether an additional cofactor was needed by the strain. The requirement for supplements to the mineral medium was also described for other *Roseobacter *clade bacteria [93,94]. The cultures were inoculated (1% v/v) with cells grown in liquid complex medium.

For the tests of nitrogen and sulfur sources, mannitol was used as carbon source for *R. litoralis *and glycerol for *R. denitrificans*, as growth of the organisms with these substrates yielded similar optical densities and no addition of complex medium was necessary to support growth. As inoculum for the nitrogen tests 200 μL (4% v/v) of N-starved stationary phase cultures were used. All algal osmolytes that *R. litoralis *could use as carbon and nitrogen sources in separated experiments were additionally tested combined as carbon and nitrogen source within one experiment with a final concentration of 10 mM. In all tests, inoculated parallels that contained no carbon, nitrogen or sulfur source, respectively, served as negative controls. Growth was considered as positive if the optical density was higher than in the respective negative controls. All cultures were incubated at room temperature with a natural day-night-rhythm. At suitable time intervals the optical density at 600 nm (OD_600_) was measured with a spectrophotometer (Bausch & Lomb). After reaching the stationary phase, cells were transferred into fresh medium containing the respective substrate to confirm growth. When taurine served as sulfur source, the cells were transferred twice to fresh medium because the sulfur-free cultures were not growth limited compared to the parallels with taurine in the first two passages. After reaching the stationary phase in the second growth passage, purity of each culture was tested on an agar plate.

### Intracellular glycogen

*R. litoralis *and *R. denitrificans *were tested for intracellular production of glycogen when grown in complex medium (modified 70% MB, see Additional File 5) and in mineral medium using the method described by Bourassa and Camilli [57]. As mineral medium, modified MBM with NaHCO_3 _as buffer, 5 mM ammonium and 1 mg/mL mannitol as carbon source (1 mL/L glycerol for *R. denitrificans*) was used. After growth the cells were pelleted, washed once with 1 × PBS (phosphate buffered saline) buffer and stored frozen until further processing.

### Nucleotide sequence accession number

The complete genome sequence of *Roseobacter litoralis *OCh149 was deposited in GenBank under the accession numbers [GenBank:CP002623]-[GenBank:CP002626].

## Authors' contributions

DK performed the genome analysis, the experimental work and drafted the manuscript. ST analysed the genomic islands and helped to draft the manuscript. SV supervised the genome finishing, did the comparative studies, helped with the island prediction and was involved in drafting the manuscript. RL submitted the sequence data. HL did the assemblies of sequence data and helped with the finishing and annotation of the genome. AW provided the BiBag tool and helped with the BioCyc database for pathway reconstruction. RD and MS conceived of the study and helped to draft the manuscript. TB conceived of the study, supervised the experimental work and was involved in drafting the manuscript. All authors read and approved the final manuscript.

## Supplementary Material

Additional file 1 **Genes with assigned function that distinguish *R. litoralis *and *R. denitrificans***. Click here for file

Additional file 2 ***rfb*-genes (antigen biosynthesis) in the genomes of marine *Roseobacter *clade members**. The isolation sources are according to the information given in the IMG database [38]. Marked in blue are the exceptions, i.e. the organisms that were isolated from hosts but have chromosome-located *rfb*-genes and the pelagic organisms with plasmid-located *rfb*-genes. Click here for file

Additional file 3 **Comparison of the photosynthetic gene clusters of different anoxygenic phototrophs**. The data for *H. phototrophica *DFL-43 and *L. alexandrii *DFL-11 are based on the draft genome sequences. The gene organization of *R. litoralis *and *R. denitrificans *is identical, as is the case for *H. phototrophica *and *L. alexandrii*. The gene organization of *D. shibae *differs from the other two types. The two *Roseobacter *species show a similar, growth phase dependent response to light. *H. phototrophica *and *L. alexandrii *are not closely related but have a similar regulation of bacteriochlorophyll-*a *production, whereas the regulation mechanism of *D. shibae *is different [4]. Therefore, the gene organization and the location of the regulators may be important for the global regulation of the photosynthetic activity in aerobic anoxygenic phototrophic bacteria. Click here for file

Additional file 4 **Results from substrate tests with *R. litoralis *and predictions of substrate utilization from the genome**. In bold are the substrates for which experimental and genomic data differ. -, OD600 equal or less than negative control; +, OD600 < 0.2; ++, OD600 0.2 - 0.5; +++, OD600 > 0.5; w, negative control < OD600 < 0.2. Results are shown for the second growth passage unless otherwise indicated. All amino acids had L-conformation, all sugars had D-conformation unless otherwise indicated. Growth on almost all sugars and sugar derivatives was considerably enhanced by the addition of 1% complex medium to the mineral medium. Glucose was not utilized without this supplementation. Addition of complex medium did not enhance the growth of *R. litoralis *on most amino acids. Exceptions were glutamate and the amino sulfonic acid taurine which were not utilized without supplementation. Click here for file

Additional file 5 **Composition of Culture Media**. Click here for file
