# Supplementary material for: Comparative genome analysis and genome-guided physiological analysis of Roseobacter litoralis
Source: BMC Genomics. 2011 Jun 21;12:324. doi: 10.1186/1471-2164-12-324 (PMC3141670; doi:10.1186/1471-2164-12-324)
Supplement: Additional file 3 — Comparison of the photosynthetic gene clusters of different anoxygenic phototrophs. The data for H. phototrophica DFL-43 and L. alexandrii DFL-11 are based on the draft genome sequences. The gene organization of R. litoralis and R. denitrificans is identical, as is the case for H. phototrophica and L. alexandrii. The gene organization of D. shibae differs from the other two types. The two Roseobacter species show a similar, growth phase dependent response to light. H. phototrophica and L. alexandrii are not closely related but have a similar regulation of bacteriochlorophyll-a production, whereas the regulation mechanism of D. shibae is different [4]. Therefore, the gene organization and the location of the regulators may be important for the global regulation of the photosynthetic activity in aerobic anoxygenic phototrophic bacteria. [file 1471-2164-12-324-S3.PDF]

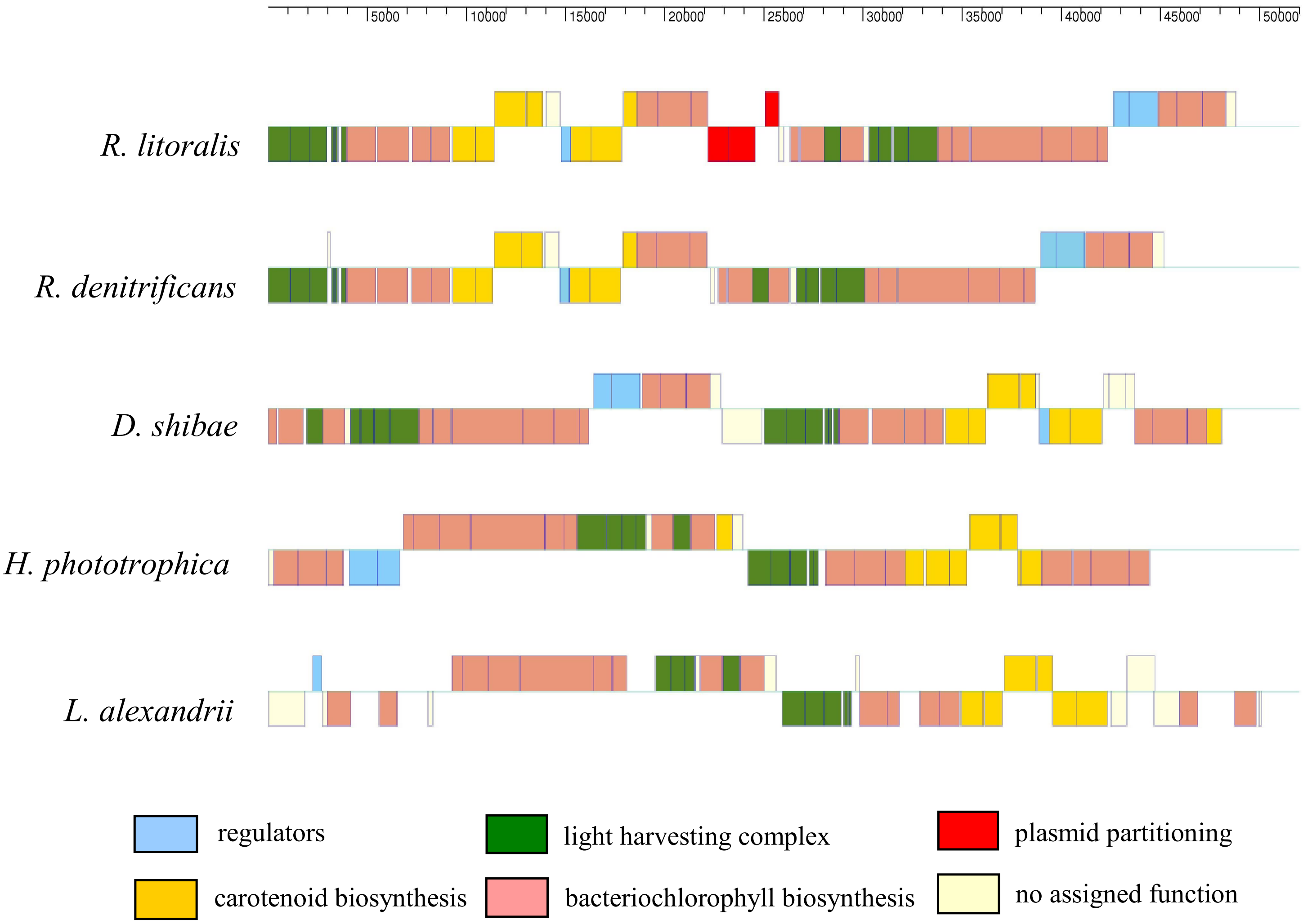

Additional File 3 Comparison of the photosynthetic gene clusters of different anoxygenic phototrophs. The data for *H. phototrophica* DFL-43 and *L. alexandrii* DFL-11 are based on the draft genome sequences. The gene organization of *R. litoralis* and *R. denitrificans* is identical, as is the case for *H. phototrophica* and *L. alexandrii*. The gene organization of *D. shibae* differs from the other two types. The two *Roseobacter* species show a similar, growth phase dependent response to light. *H. phototrophica* and *L. alexandrii* are not closely related but have a similar regulation of bacteriochlorophyll-*a* production, whereas the regulation mechanism of *D. shibae* is different [1]. Therefore, the gene organization and the location of the regulators may be important for the global regulation of the photosynthetic activity in aerobic anoxygenic phototrophic bacteria.

1. Holert J, Hahnke S, Cypionka H: **Influence of light and anoxia on chemiosmotic energy conservation in *Dinoroseobacter shibae***. *Environ Microbiol Rep* 2010, **doi: 10.1111/j.1758-2229.2010.00199.x**.
