# Supplementary material for: Comparative genome analysis and genome-guided physiological analysis of Roseobacter litoralis
Source: BMC Genomics. 2011 Jun 21;12:324. doi: 10.1186/1471-2164-12-324 (PMC3141670; doi:10.1186/1471-2164-12-324)
Supplement: Additional file 5 — Composition of Culture Media. [file 1471-2164-12-324-S5.PDF]

Additional File 5: Composition of culture media

70 % Marine Broth (MB, Difco 2216), modified, 1 L, pH 7.5

8.82 g $MgCl_2$ x 6 H_2O, 2.27 g Na_2SO_4, 13.5 g NaCl, 1.67 g, $CaCl_2$ x 2 H_2O, 0.385 g KCl, 0.112 g $NHCO_3$, 0.007 g Na_2HPO_4 x 2 H_2O, 0.1 g ferric citrate, 1 g yeast extract, 1 g peptone, 1 g soytone, 1 mL glycerol, 7 mL trace element stock solution.

Trace element stock solution, 500 mL

4.0 g KBr, 2.85 g $SrCL_2$ x 6 H_2O, 1.06 g, H_3BO_3, 0.35 g Na-Silicat x 5 H_2O, 0.12 g NaF, 0.08 g NH_4NO_3 per 500 mL.

PPES-II medium [1], modified, 1 L, pH 7.5

700 mL artificial seawater [2], 300 mL distilled water, 2 g peptone, 1 g yeast extract, 1 g soytone, 0.1 g ferric citrate.

Agar plates, based on a medium described by Shioi et al. [3], modified, 1 L, pH 7.3

20 g NaCl, 5 g $MgCl_3$ x 6 H_2O, 2 g Na_2SO_4, 0.5 g KCl, 0.5 g $CaCl_2$ x 2 H_2O, 0.2 g $NaHCO_3$, 0.1 g ferric citrate, 1 g yeast extract, 1 g peptone, 1 g soytone, 1 mL glycerol, 14 g agar.

Marine Basal Mineral medium [4], modified, pH 7.5, 1 L

8.46 g Tris-HCl, 0.34 g NH_4Cl, 0.0022 g K_2HPO_4, 11.6 g NaCl, 12.3 g $MgSO_4$ * 7 H_2O, 0.74 g KCl, 1.46 g $CaCl_2$* 2 H_2O, 1 mL vitamin stock solution, 1 mL trace element stock solution [5].

Vitamin stock solution, 100 mL

10 mg para-aminobenzoic acid, 5 mg folic acid, 5 mg liponic acid, 10 mg riboflavin (B_2), 20 mg thiamine (B_1), 20 mg nicotinic acid amide, 50 mg pyridoxamine (B_6-amine), 10 mg panthothenic acid, 10 mg cobalamin (B_{12}), 2 mg $D^{(+)}$-biotin.

References

1. Shiba T: ***Roseobacter litoralis* gen. nov., sp. nov, and *Roseobacter denitrificans* sp. nov., aerobic pink-pigmented bacteria which contain bacteriochlorophyll-*a***. *Syst Appl Microbiol* 1991, **14**:140-145.
2. Lyman J, Fleming RH: **Composition of sea water**. *J Mar Res* 1940, **3**:134-140.
3. Shioi Y: **Growth characteristics and substrate specificity of aerobic photosynthetic bacterium, *Erythrobacter* sp. (Och 114)**. *Plant Cell Physiol* 1986, **27**:567-572.
4. Geng H, Bruhn JB, Nielsen KF, Gram L, Belas R: **Genetic dissection of tropodithietic acid biosynthesis by marine roseobacters**. *Appl Environ Microbiol* 2008, **74**:1535-1545.
